# Comparisons of maxillary incisor retraction effects for patients with first premolar extractions between Damon Q and Invisalign^®^: A retrospective study

**DOI:** 10.1097/MD.0000000000030919

**Published:** 2022-10-07

**Authors:** Jiping Chen, Juan Wen, Ling Huang, Lu Zhang, Lei Han, Huang Li

**Affiliations:** a Nanjing Stomatological Hospital, Medical School of Nanjing University, Nanjing, China; b Orthodontic Department, Nanjing Stomatological Hospital, Medical School of Nanjing University, Nanjing, China; c Stomatology Hospital, School of Stomatology, Zhejiang University School of Medicine, Clinical Research Center for Oral Diseases of Zhejiang Province, Key Laboratory of Oral Biomedical Research of Zhejiang Province, Cancer Center of Zhejiang University, Hangzhou, China.

**Keywords:** Damon Q, extraction, Invisalign^®^, maxillary incisor retraction

## Abstract

Torque control of maxillary incisors is very important in maxillary protrusion patients with first premolars extraction, but the efficacy of maxillary incisor retraction of clear aligners is still controversial now. This retrospective study was aimed to compare the retraction effects between Damon Q and Invisalign^®^ appliances in patients with first premolar extractions. 59 patients (33 cases with Damon Q and 26 cases with Invisalign^®^) with first premolar extraction were selected in this study. Subsequently, patients of each group were allocated into three subgroups according to the pretreatment value of U1-NA (°). The retraction effects of maxillary incisors and upper lips were accessed by the variations of cephalometric, overbite and overjet measurements. Treatment duration with Invisalign^®^ (31.4 ± 6.4 months) was longer than Damon Q (27.7 ± 6.3 months) (*P* = .03). The angular measurements, U1-NA (°) and U1-SN (°) showed more lingual crown inclinations in Invisalign^®^ group than Damon Q group (*P* ≤ .04). When evaluating linear measurements, the retractions of the maxillary incisors and upper lip positions showed no significant differences (*P* ≥ .13). Invisalign^®^ group also showed more lingual crown retractions and labial root deviations compared to Damon Q group in subgroup Ⅲ (*P* ≤ .037). As regards to the molar relationship, Invisalign^®^ displayed less Class Ⅰ molar relationship than Damon Q group. The increased overbite of anterior incisors was also showed in the Invisalign^®^ treatment group (*P* ≤ .047). Invisalign^®^ was not sufficiently effective in retracting maxillary incisors compared with Damon Q appliances. Invisalign^®^ led to more lingual inclination movement and increased overbite.

## 1. Introduction

Nowadays, more than 16.8% of patients seek orthodontic treatment due to protruded maxillary incisors and a convex profile.^[1–3]^ For patients with dental protrusion, the extraction of first premolars can reduce the protrusion of maxillary incisors, alleviate the incompetent upper lips, and achieve a favorable straight profile.^[2,4,5]^ Torque control of maxillary incisors is very important for achieving good results, such as anterior aesthetic. Conventional fixed appliances have been adopted as effective tools to manage the reposition of protruded incisors.^[6,7]^ Self-ligating brackets (SLBs) were designed to reduce bracket/wire friction during mechanical sliding^[8]^ and therefore facilitate alignment,^[9]^ increase intervals between activations, and reduce chairside time.^[10]^ Among SLBs, Damon Q was proven to produce the lowest friction^[11]^ and the largest corrections in the protrusion directions.^[12]^

With the technology developed, clear aligners have become increasingly common choices because of the growing number of adult patients asking for aesthetic and comfortable alternatives.^[13]^ However, the efficacy of maxillary incisor retraction of clear aligners, especially for maxillary protrusion patients, is still controversial now. Rossini et al and Li et al^[14,15]^ reported that Invisalign^®^ was not sufficiently effective in retracting maxillary incisors compared with traditional fixed appliances in 2015. Sfondrini et al^[16]^ claimed that Invisalign^®^ was as effective as self-ligating appliances (Damon Q and 3M) in incisor retraction for non-extraction patients in 2018. Dai et al^[17]^ found Invisalign^®^ resulted in more teeth inclination and less retraction than predicted for maxillary central incisors in 2019.

To investigate the maxillary incisors retraction effect of Invisalign^®^ and Damon Q systems, patients with first premolars extraction were included in this retrospective study. Treatment effects were evaluated with the change of cephalometric, overbite and overjet measurements. The crown and root retraction of maxillary central incisors and upper lips position change were further accessed in three subgroups according to the pretreatment maxillary incisors’ inclination. The result from our study should more evidence for Invisalign^®^ treatment in the maxillary protrusion patients with first premolars extraction.

## 2. Materials and methods

**Figure 1.**
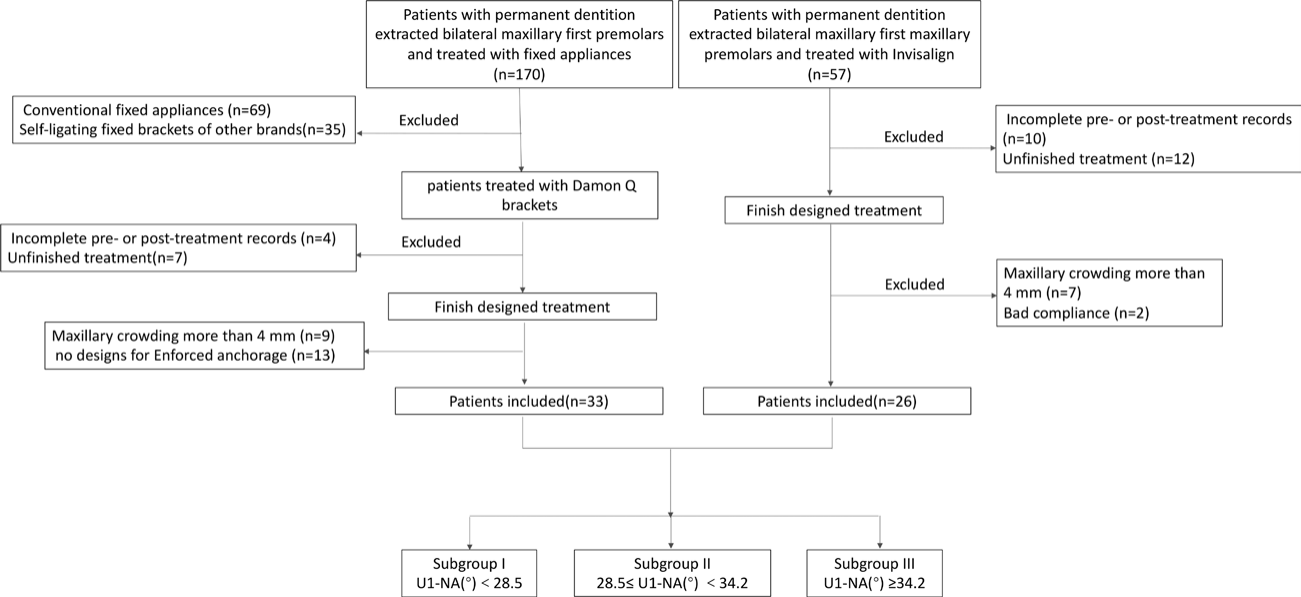
The sampling flow chart.

Our research protocol was ethically approved by the Ethical Standards of Nanjing Stomatological Hospital, Medical School of Nanjing University (NJSH-2021NL-87). Sixty-one patients from the Orthodontic Department of Nanjing Stomatological Hospital, Medical School of Nanjing involved in this study (Fig. 1). Informed consent was obtained from each participant. Thirty-three patients were treated with Damon Q, and twenty-six were treated with Invisalign^®^.

### 2.1. Patients and eligibility criteria

Patient eligibility criteria: ANB (°): 2-8 and U1-NA (°): ≥ 22.8; Extraction of bilateral maxillary first premolars and mandibular first or second premolars; Mild maxillary crowding (<4 mm); and Complete records. Exclusion criteria: Craniofacial defects, syndromes or skeletal deformities; History of trauma on the maxillary teeth; and History of orthodontic treatment.

In order to analyze the retraction effects detailly, all patients were allocated into 3 subgroups according to the protrusion degree of maxillary incisors. The allocation was based on the normal mean (22.8) and standard deviation (5.7) obtained for U1-NA (°). Subgroup I: 22.8 ≤ U1-NA (°) < 28.5 (Mean + 1 SD); Subgroup II: 28.5 (Mean + 1 SD) ≤ U1-NA (°) < 34.2 (Mean + 2 SD); Subgroup III: U1-NA (°) ≥ 34.2 (Mean + 2 SD). The sampling process is shown in Figure 1.

### 2.2. Treatment design

Damon Q: 0.022 × 0.028-inch slot brackets with standard prescription torque (+15° and +6° for maxillary central and lateral incisors, respectively) and 0.019 × 0.025-inch stainless steel wire were used to retract the maxillary incisors. Transpalatal arches and maxillary second molars were included to reinforce anchorage.

Invisalign^®^: All aligners (Align Technology, San Jose, CA) were designed and manufactured under the G6 protocol. Distal bodily movement of the canines was followed by the inclination of the maxillary anterior teeth. The torque expression was accomplished by Power Ridge technology. The Invisalign^®^ was instructed for wearing more than 22 hours a day. All patients were guided to change each aligner for two weeks and use chewies every day.

### 2.3. Lateral cephalometric radiographs

Lateral cephalometric radiographs standardized with head posture and maximal intercuspation were taken by an X-ray cephalograph (ORTHOCEPH OC200 D; Instrumentarium Dental, Tuusula, Finland). The computerized cephalometric measurements were performed using Dolphin Imaging software (Dolphin Imaging & Management Solutions, 11.5, Oakdale, CA).

U1-NA (°), U1-NA (mm), U1-SN (°) and maxillary lip to E-plane (mm) were selected to evaluate the maxillary incisors retraction effect of Damon Q and Invisalign^®^. The root retractions of maxillary central incisors were evaluated with the distance to the NA plane of apical point (U1R-NA (mm)). If the variation of U1R-NA (mm) is larger than 0, it means the root has been lingually retracted. If the variation of U1R-NA (mm) shows negative numbers, it means the root moved labially.

The measurements were performed on all of the images independently by two investigators (Jiping Chen and Lu Zhang). Intraclass correlation coefficient (ICC) tests were used to assess the intraoperator and interoperator errors. The average ICC for interrater reliability was 0.88, and the average ICCs for intrarater reliability were 0.93 and 0.94 for the two investigators respectively.

### 2.4. Statistical analysis

Statistical analysis was performed with SPSS^®^ Statistics 24 software (SPSS Statistics, version 24.0; IBM, Armonk, NY). The normality and homogeneity of the data were tested with the Shapiro–Wilk and Levene tests. The chi-square test was used to evaluate the distributions of age and sex in each group. Differences between the two groups with respect to skeletal pattern, labiolingual inclination of the maxillary central incisors, and facial profile before and after orthodontic treatment as well as their variations were compared by Independent samples *t* test. The Mann–Whitney *U* test was adopted if normality or homogeneity of variables was not met. Multiple comparisons of the cephalometric measurements among subgroups were performed by ANOVA with the LSD post hoc test. The Kruskal–Wallis test with the Nemenyi post hoc test was adopted if normality or homogeneity of variables was not met. For all tests, the significance level was set at *P* < .05.

## 3. Results

As shown in Tables 1 and 2, the demographic distributions and cephalometric measurements (T0) before treatment showed no significant difference (*P* ≥ .06) between Damon Q and Invisalign^®^ groups, which confirms the two groups were matched for age, sex and malocclusion. Compared with 27.7 ± 6.3 months for Damon Q group, longer treatment duration was obtained for Invisalign^®^ group (31.4 ± 6.4 months) (*P* = .03) (Table 1).

**Table 1:** Demographic composition of the study sample.

	Damon Q (n = 33)	Invisalign^®^ (n = 26)	*P* value
Age (yr)	21.4 ± 6.6	23.8 ± 7.1	.20
Gender			
Male	7	5	.85
Female	26	21	
Treatment duration (mo)	27.7 ± 6.3	31.4 ± 6.4	.03

The age and sex distribution for Damon Q and Invisalign^®^ were matched and showed no significant difference (*P* = .20). The treatment duration of Invisalign^®^ was 31.4 ± 6.4 mo, statistically longer than Damon Q (*P* = .03). Age was expressed in ages and treatment duration was expressed in months. Independent *t* test and chi-square test were used separately to evaluate the distribution of different parameters between Damon Q and Invisalign^®^.

**Table 2:** Descriptive statistics of pretreatment (T0), post-treatment (T1) and treatment variations (T0-T1).

	Damon Q			Invisalign^®^			Significance between				
	T0①	T1②	T0-T1③	T0④	T1⑤	T0-T1⑥	①④	②⑤		③⑥	
SNA (°)	82.00 ± 3.64	81.49 ± 3.60	0.51 ± 0.81	82.94 ± 2.88	82.39 ± 2.53	0.55 ± 2.07	0.15*		0.29		0.84*
SNB (°)	76.59 ± 3.32	76.36 ± 3.37	0.23 ± 0.91	78.79 ± 4.71	77.38 ± 3.26	1.41 ± 3.20	0.08		0.24		0.19*
ANB (°)	5.41 ± 2.05	5.17 ± 1.95	0.24 ± 0.84	4.91 ± 2.04	5.02 ± 1.76	−0.10 ± 1.87	0.97*		0.76		0.33*
MP-SN (°)	35.77 ± 7.11	35.89 ± 7.09	−0.12 ± 2.00	37.33 ± 8.82	36.71 ± 7.08	0.62 ± 5.36	0.69*		0.66		0.96*
Y-axis (°)	72.13 ± 3.83	71.96 ± 5.33	0.17 ± 3.11	72.82 ± 5.63	73.00 ± 4.40	−0.18 ± 2.91	0.78*		0.97*		0.97*
MP-FH (°)	29.87 ± 7.54	30.02 ± 7.68	−0.15 ± 4.21	32.64 ± 9.53	30.77 ± 8.82	1.87 ± 8.16	0.32*		0.73		0.99*
U1-NA (°)	32.29 ± 5.03	15.92 ± 6.25	16.38 ± 5.22	34.76 ± 7.51	14.26 ± 6.12	20.50 ± 7.88	0.25		0.31		0.03
U1-NA (mm)	8.10 ± 1.80	2.50 ± 2.26	5.59 ± 2.28	9.19 ± 2.51	2.47 ± 2.23	6.72 ± 2.41	0.06		0.96*		0.13*
U1R-NA (mm)	4.21 ± 1.59	3.85 ± 1.50	−0.35 ± 1.35	4.14 ± 2.40	3.21 ± 1.90	−0.98 ± 2.01	0.96		0.15		0.16
U1-SN (°)	114.29 ± 5.38	97.44 ± 6.35	16.85 ± 5.23	117.68 ± 8.11	96.66 ± 6.82	21.02 ± 8.62	0.07		0.65		0.04
Maxillary Lip to E-plane (mm)	1.95 ± 2.23	-0.70 ± 2.61	2.02 ± 1.51	2.04 ± 2.64	-0.32 ± 1.68	2.36 ± 2.11	0.88		0.66		0.47

There was no significant difference between two groups in skeletal, dental and soft tissue parameters in pretreatment (T0). More lingual crown inclinations were obtained in Invisalign^®^ group compared to Damon Q group (*P* ≤ .04) when evaluating angular variations of U1-NA (°) and U1-SN (°). Linear retractions of the maxillary incisors and upper lip positions for both groups showed no significance (*P* ≥ .13).

* Independent *t* test and Mann–Whitney *U* test were used.

### 3.1. Comparisons of cephalometric measurements pre- and post-treatment for Damon Q and Invisalign^®^ group

**Figure 2.**
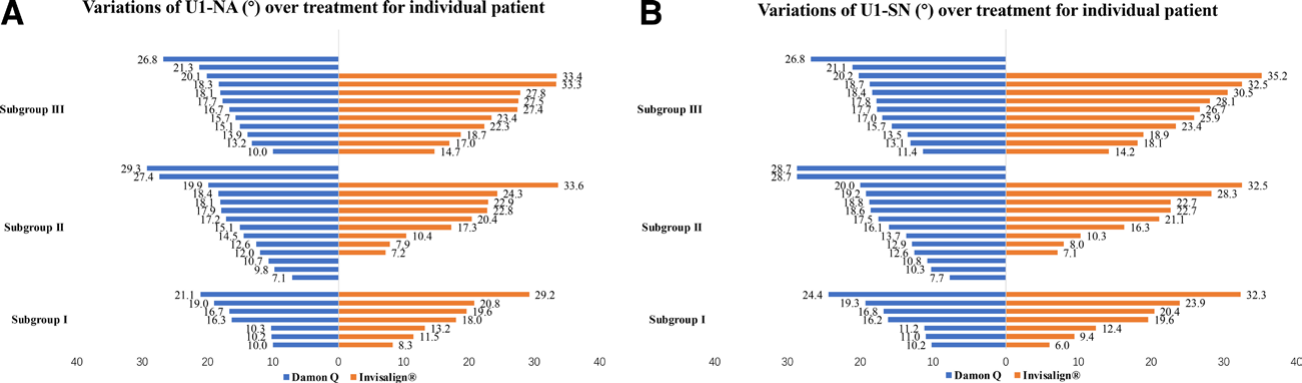
Variations of U1-NA (°) and U1-SN (°) over treatment for individual patient. The variations of U1-NA (°) (A) and U1-SN (°) (B) with Damon Q ranged from 29.3° to 7.1°and 28.7° to 7.7°. The variations of U1-NA (°) and U1-SN (°) with Invisalign^®^ ranged from 33.6° to 7.2°and 35.2° to 6.0°.

The pretreatment skeletal, dental and soft tissue parameters for Damon Q and Invisalign^®^ group displayed no significance (Table 2). When evaluating angular variations of U1-NA (°) and U1-SN (°), Invisalign^®^ group showed more lingual crown inclinations than Damon Q group (*P* ≤ .04) (Table 2). The inclination changes of U1-NA (°) ranged from 29.3° to 7.1° and from 33.6° to 7.2° in Damon Q and Invisalign^®^ group respectively (Fig. 2A). The inclination changes of U1-SN (°) ranged from 28.7° to 7.7°and from 35.2° to 6.0° in Damon Q and Invisalign^®^ group respectively (Fig. 2B). Damon Q and Invisalign^®^ group showed no significance in linear retractions of the maxillary incisors and upper lip positions (*P* ≥ .13) (Table 2).

### 3.2. Subgroup comparisons between Damon Q and Invisalign^®^ group

**Figure 3.**
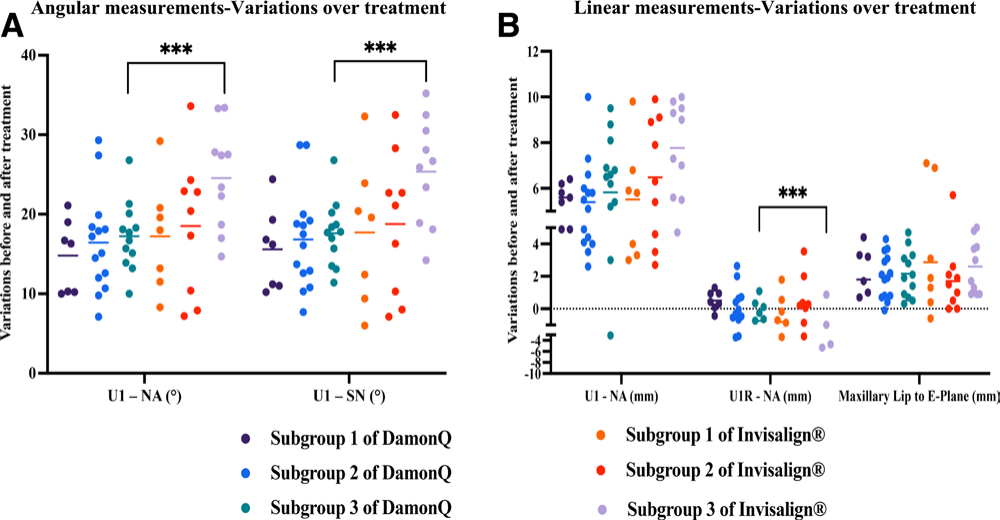
Angular and linear variations over treatment in different subgroups for individual patient. Invisalign^®^ resulted in more lingual crown inclinations (A, U1-NA (°) and U1-SN (°)) and buccal root deviations (B, U1R-NA (mm)) compared with Damon Q in subgroup Ⅲ (*P* ≤ .04). Independent *t* test (Mann–Whitney *U* test) and ANOVA (Kruskal–Wallis test) were applied. ***P < 0.001.

The individual change for the 5 tested angular and linear variables was showed in Figure 3 and Table 3. In subgroup Ⅲ, more retractions were obtained with Invisalign^®^ compared to Damon Q (*P* ≤ .001, Table 3), and more labial root deviations were found with Invisalign^®^ than Damon Q in subgroup Ⅲ (*P* = .04, Table 3). However, maxillary Lip to E-plane (mm) distance did not show significant differences among 3 subgroups between Damon Q and Invisalign^®^ groups (Table 3).

**Table 3:** Descriptive statistics of 3 subgroups T0-T1.

	Damon Q (Mean ± SD)			Invisalign^®^ (Mean ± SD)			Significance between				
	Subgroup I ① (n = 7)	Subgroup II ② (n = 14)	Subgroup III ③ (n = 12)	Subgroup I ④ (n = 7)	Subgroup II ⑤ (n = 9)	Subgroup III ⑥ (n = 10)	①②③	④⑤⑥	①④	②⑤	③⑥
U1-NA (°)	14.80 ± 4.61	16.43 ± 6.27	17.56 ± 3.88	17.23 ± 6.96	18.53 ± 8.73	24.55 ± 6.47	0.63	0.11	0.46	0.51	0.01
U1-NA (mm)	5.60 ± 0.59	5.40 ± 1.83	5.07 ± 3.27	5.51 ± 2.37	6.48 ± 2.61	7.77 ± 2.00	0.84*	0.16	0.93	0.26	0.09†
U1R-NA (mm)	0.51 ± 0.59	-0.44 ± 1.76	-0.74 ± 0.90	-0.82 ± 1.83	0.11 ± 1.93	-2.06 ± 1.79	0.14	0.06	0.09	0.49	0.04
U1-SN (°)	15.59 ± 5.21	16.83 ± 6.27	17.98 ± 3.92	17.71 ± 9.10	18.78 ± 8.98	25.35 ± 6.72	0.73	0.12	0.60	0.55	0.00
Maxillary Lip to E-plane (mm)	1.80 ± 12.04	2.01 ± 1.37	2.23 ± 1.52	2.87 ± 3.05	1.70 ± 1.76	2.60 ± 1.65	0.90	0.51	0.46	0.64	0.51

Similar retraction effect among the 3 subgroups were obtained in both Damon Q and Invisalign^®^ groups (*P* ≥ .06). More retractions and more labial root deviations were also obtained with Invisalign^®^ in subgroup Ⅲ (*P* ≤ .04). Maxillary Lip to E-plane (mm) distance did not show significant differences among 3 subgroups between Damon Q and Invisalign^®^ groups.

* Multiple comparisons of 3 subgroups for Damon Q or Invisalign^®^ were tested with ANOVA or Kruskal–Wallis test.

† Comparisons between Damon Q and Invisalign^®^ in different subgroups were tested with Independent *t* test or Mann–Whitney *U* test.

### 3.3. Comparisons of dental measurement pre- and post-treatment for Damon Q and Invisalign^®^ group

The changes of molar relationship and anterior overjet and overbite were showed in Table 4. 69.2% patients (18/26) in Invisalign^®^ group obtained a Class I molar relationship, which was less than 87.8% (29/33) in Damon Q group (*P* = .012). Damon Q also displayed higher capacity to improve anterior overbite than Invisalign^®^ (*P* = .047). The percentage of normal overbite in patients was increased from 36.3% to 90.9% in Damon Q group after treatment, while from 42.3% to 61.5% in Invisalign^®^ group. The difference of overjet improvement was no significant between Damon Q and Invisalign^®^ group.

**Table 4:** Malocclusion improvements in Damon Q and Invisalign^®^ group.

		Damon Q (n = 33)		Invisalign^®^ (n = 26)		*P* value
		T0	T1	T0	T1	
Angle classification						
Class I		13	29	17	18	.012
Class II		19	4	8	8	
Class III		1	0	1	0	
Anterior overjet						
Normal		5	31	3	24	.373
Deep overjet	I°	6	2	8	2	
	II°	10	0	9	0	
	III°	12	0	6	0	
Anterior overbite						
Normal		12	30	11	16	.047
Deep overbite	I°	8	2	8	7	
	II°	9	1	3	3	
	III°	4	0	4	0	

Damon Q achieved more improvements on molar relationship and overbite than Invisalign^®^ (*P* ≤ .047). The percentage of Class I molar relationship and normal overbite in patients was increased from 39.4% to 87.8% and 36.3% to 90.9% in Damon Q group after treatment. The percentage of Class I molar relationship and normal overbite in patients was slightly increased from 65.4% to 69.2% and 42.3% to 61.5% in Invisalign^®^ after treatment. The difference of overjet improvement was no significant between two groups after treatment. Angle classification, Overjet and Overbite were ranked orthodontic data. The calculated rank of data (T0-T1) was analyzed with Mann–Whitney *U* test.

## 4. Discussion

The retraction of the maxillary incisors is crucial for the acquisition of a favorable profile in orthodontic treatment. Fixed appliances such as Damon Q SLBs have been demonstrated as effective tools to retract protruded incisors. In recent years, clear aligners have become an alternative option for fixed appliances but their management for maxillary incisors case has not been explored very clearly, especially in extraction cases. The retrospective study was performed to investigate the maxillary incisors retraction effect of Invisalign^®^ and Damon Q systems for patients with first premolars extraction.

We found that Invisalign^®^ was not sufficiently effective in retracting maxillary incisors compared with Damon Q appliances. This was similar to the previous studies.^[14,15]^ Although the SmartTrack^®^ material and G6 protocol were proposed in Invisalign^®^, the torque control of maxillary incisors still be a challenge for Invisalign^®^. Compared with Damon Q, more maxillary incisors lingual inclination changes were recorded from T0-T1, and especially apparent in the subgroup Ⅲ, indicating more tipping movements for severely protruded maxillary incisors. The previous report^[17–19]^ also have the similar findings, as more labial root deviation and insufficient torque expression with Invisalign^®^ when applied in extraction cases. Meanwhile, the more labial root deviation with Invisalign^®^ also indicated a higher risk of creating or exacerbating bone fenestration during orthodontic treatment^[20]^ although Invisalign^®^ used to be considered good for periodontal health.^[21]^ The last thing we need pay attention to the longer treatment duration in the Invisalign^®^ group, which may explain by the higher cooperation requirement with removable clear aligner.

According to the improvement of molar relationship and anterior overjet and overbite, Damon Q group displayed larger portion of patients with Class I molar relationship and normal overbite than Invisalign^®^ group after treatment. This suggested Invisalign^®^ was not sufficiently in retracting the maxillary incisors, which showed limited ability to correct sagittal and anterior vertical discrepancies.^[22]^ Systemic review and clinical study also reported the intrusion of maxillary incisors and correction of deep overbite with Invisalign^®^ were insufficient,^[23–25]^ which may resulting from the poor expression of prescribed overbite reduction (no more than 39.2%) of Invisalign^®^ as reported by Blundell et al.^[25]^

There are still several limitations in this study. The sample size of this study was not very large. The parameters obtained by 2D radiographs for measuring the inclinations of the maxillary incisors may not be very precise. Further studies by three-dimensional analysis are needed in the future.

## 5. Conclusion

- In patients with bilateral maxillary first premolar extractions, Invisalign^®^ was not sufficiently effective in retracting maxillary incisors compared with Damon Q appliances.
- Invisalign^®^ caused more lingual inclination of maxillary incisors in patients with bilateral maxillary first premolar extraction, especially those with severely protruded maxillary incisors.

## Acknowledgments

We would like to thank Miss Chongchong Zhou from Department of Research Management, Nanjing Stomatological Hospital, Medical School of Nanjing University, for the statistical consultation of this study.

## Author contributions

**Conceptualization:** Huang Li.

**Data curation:** Jiping Chen. Juan Wen, Ling Huang, Lu Zhang, Lei Han.

**Formal analysis:** Jiping Chen.

**Supervision:** Huang Li.

**Writing – original draft:** Jiping Chen.

**Writing – review & editing:** Jiping Chen, Juan Wen, Huang Li.
